# Pharyngeal bulb prosthesis and speech outcome in patients with cleft palate

**DOI:** 10.1016/j.bjorl.2020.05.028

**Published:** 2020-07-21

**Authors:** Maria Inês Pegoraro-Krook, Raquel Rodrigues Rosa, Homero C. Aferri, Laura Katarine Félix de Andrade, Jeniffer de C.R. Dutka

**Affiliations:** a Universidade de São Paulo, Faculdade de Odontologia de Bauru, Departamento de Fonoaudiologia, Bauru, SP, Brazil; b Universidade de São Paulo, Hospital de Reabilitação de Anomalias Craniofaciais, Serviço de Prótese de Palato, Bauru, SP, Brazil; c Universidade de São Paulo, Hospital de Reabilitação de Anomalias Craniofaciais, Programa de Pós-Graduação em Ciências da Reabilitação, Bauru, SP, Brazil

**Keywords:** Cleft palate, Velopharyngeal insufficiency, Speech, Palatal obturator

## Abstract

**Introduction:**

Individuals with cleft palate can present with velopharyngeal dysfunction after primary palatoplasty and require a secondary treatment due to insufficiency. In these cases, the pharyngeal bulb prosthesis can be used temporarily while awaiting secondary surgery.

**Objective:**

This study aimed to investigate the outcome of treatment of hypernasality with pharyngeal bulb prosthesis in patients with history of cleft palate presenting with velopharyngeal insufficiency after primary palatal surgery. We hypothesized that the use of the pharyngeal bulb prosthesis is an effective approach to eliminate hypernasality related to velopharyngeal insufficiency in patients with cleft palate.

**Methods:**

Thirty speakers of Brazilian Portuguese (15 males and 15 females) with operated cleft palate, ages ranging from 6 to 14 years (mean: 9 years; SD = 1.87 years), participated in the study. All patients were fitted with a pharyngeal bulb prosthesis to manage velopharyngeal insufficiency while they were awaiting corrective surgery to be scheduled. Auditory-perceptual analysis of speech recorded in the conditions with and without pharyngeal bulb prosthesis were obtained from three listeners who rated the presence or absence of hypernasality for this study.

**Results:**

Seventy percent of the patients eliminated hypernasality while employing the pharyngeal bulb prosthesis, while 30% still presented with hypernasality. The comparison was statistically significant (*p* < 0.001).

**Conclusion:**

The use of the pharyngeal bulb prosthesis is an effective approach to eliminate hypernasality related to velopharyngeal insufficiency.

## Introduction

Adequate velopharyngeal mechanism (VPM) functioning is essential for normal speech production.^1^ Cleft palate is an anatomical defect usually repaired during the first year of life. The goals of cleft palate repair include establishing conditions for adequate functioning of the VPM. The primary repair of cleft palate, therefore, is essential to prevent speech disorders related to oronasal coupling and also to minimize or eliminate nasal regurgitation and middle ear conditions related to auditory tube dysfunction.^2^, ^3^ Despite scientific advances, velopharyngeal dysfunction (VPD) following cleft palate primary repair occurs in 5%–43% of the cases, resulting in the inability to completely separate the oral and nasal cavities during speech.^4^, ^5^, ^6^, ^7^, ^8^, ^9^

In the population with the history of cleft palate, VPD can be characterized by velopharyngeal insufficiency (VPI), when the velum is too short or does not stretch sufficiently to reach the posterior pharyngeal wall, and velopharyngeal (VP) mislearning, when the speaker has poor or no movement of the velum and pharyngeal walls (without neurogenic etiology). Hypernasality and nasal air emission, as well compensatory articulation, are the speech symptoms most commonly associated to VPD, which can impair intelligibility.^10^, ^11^

VPD may be treated by surgery, prosthesis, speech therapy, or a combination of these approaches. Although surgery is the most common choice to treat VPI, it may not be possible or practical in some conditions, particularly when VPI co-occurs along with velopharyngeal mislearning for speech. The pharyngeal bulb prosthesis may be the best choice when surgery is contraindicated due to systemic, anatomic, or functional limitations, or when the patients choose not undergo surgery. It can be used permanently (if the patient will never undergo surgery) or temporarily when surgery needs to be delayed for medical reasons or due to the rehabilitation process itself.^12^ The bulb can also be used as a diagnostic tool when surgical prognosis is not clear,^13^, ^14^ and as a tool (associated with speech therapy) to increase movement of the VP structures to improve the prognosis of the surgical correction of VPI.^15^, ^16^ In our clinical practice, we also recommend the temporary use of a pharyngeal bulb (combined with intensive speech therapy) for patients presenting with hypodynamic velopharynx. The hypodynamic velopharynx for speech involves “the concurrence of a residual VP (Velopharyngeal) endoscopic or fluoroscopic gap size, or both forms, during attempts at maximal closure greater than 50% of the resting VP space, and the endoscopic observation of feeble velopharyngeal motion”.^17^ Individuals with a hypodynamic velopharynx, usually present with insufficiency (structural cause for the VPD) and mislearning (functional cause for the VPD), therefore need a combination of physical treatment (surgical or prosthetic) and speech therapy.^16^

The pharyngeal bulb prosthesis is a prosthetic device composed of three parts: the anterior, which is similar to a dental retainer (removable partial denture, complete denture, overdenture or an acrylic plate), the intermediate (connects the anterior part to the bulb), and the bulb (posterior part), which is customized to fill the VP gap during speech oral sounds. The bulb aims to promote the functional competence of the VPM when the lateral pharyngeal walls are able to touch the bulb during speech.^18^

The favorable results of the pharyngeal bulb prosthesis, as well as the secondary surgery depend on the amount of movement of the pharyngeal walls and the velum.^10^ Studies suggested that the movements of the pharyngeal walls can be increased with the use of a pharyngeal bulb prosthesis, with reports of a considerable decrease in the VP gap size.^10^, ^15^, ^16^, ^19^, ^20^, ^21^ However, it is still not well understood how the bulb can contribute to increase the movement of the pharyngeal walls. It is likely that the bulb acts as a sensorimotor stimulator that could facilitate muscle function, especially when its use is associated with intensive speech therapy.^22^, ^23^, ^24^ Voluntary modification of VP movement therapeutically is not an easy task since it is not possible to regulate proprioception of the velopharynx. Some type of “feedback” (auditory, tactile) that increases information regarding VP activity during speech might be triggered by the presence of the bulb filling the VP gap.^25^

The use of the temporary pharyngeal bulb (combined or not with speech therapy) to manipulate patterns of VP functioning prior to surgical correction of VPI may favor the correction of cleft palate speech for patients of all ages, including children.^10^, ^26^, ^27^, ^28^, ^29^, ^30^ Choosing a tailor-made pharyngeal bulb (that provides the possibility of VP closure) guided to elicit and improve displacement of the pharyngeal walls and velum requires should be done cautiously. Fitting a pharyngeal bulb is particularly challenging for children. The dentist has to deal not only with the challenges inherent in the preparation of any dental prosthetic procedure in a child (hygiene, prosthesis care, collaboration, etc.), but also with the challenge of making a prosthesis that the child will use during the period of facial growth considering changes in the stages of dentition, and the need for orthodontic treatment, alveolar bone graft surgery, etc.^28^, ^31^

Pharyngeal bulb use combined with speech therapy has been shown to reduce hypernasality and to improve speech intelligibility.^21^, ^32^, ^33^ However, the studies investigating the speech results of patients wearing pharyngeal bulbs (combined with speech therapy or not) were done with small sample sizes and usually reported descriptive statistics only. None has yet investigated the effect of a temporary pharyngeal bulb on the speech of cleft palate children. This study aimed to investigate the outcome of treatment of hypernasality with pharyngeal bulb prosthesis in patients with history of cleft palate presenting with velopharyngeal insufficiency after primary palatal surgery. We hypothesized that the use of the pharyngeal bulb prosthesis is an effective approach to eliminate hypernasality related to velopharyngeal insufficiency in patients with cleft palate.

## Methods

The study was approved the Institutional Review and Ethical Board of the Hospital for Rehabilitation of Craniofacial Anomalies at University of São Paulo - HRAC/USP (179/2009 - SVAPEPE-CEP). The Speech Appliances Services of HRAC/USP maintains a database of audio recordings of patients receiving prosthetic treatment of VPD. All pre-existing audio speech recordings obtained in the conditions with and without pharyngeal bulb prosthesis were considered for this study.

### Participants

The selection of speech recordings for this study was carried out prospectively, using convenience sampling. The recordings included were obtained from Brazilian Portuguese speakers with history of cleft palate who underwent temporary prosthetic treatment of VPI. Prosthetic treatment was indicated for this population instead of surgical repair of VPI due to the necessity for them to stay in a waiting line for surgery. Waiting line for secondary surgeries is a reality in our hospital, although considerable efforts that have been made to understand and improve the management of waiting lists. We are a free-cost public hospital with great caseload and demand.

The recordings studied were obtained during the patients’ continuity care visit to the Speech Appliances Service of the Institution. The following inclusion criteria was applied and the speech recordings studied were obtained from speakers with: 1) Non- syndromic operated cleft palate (with or without cleft lip) without palatal fistula; 2) Age between 6 and 14 years; 3) Normal hearing (25 dB or better hearing threshold bilaterally evaluated by an audiologist using pure tone audiometry); 4) No cognitive or neuromotor disorders; 5) Diagnosis of VPI after primary palatal surgery; 6) Use of pharyngeal bulb prosthesis for treatment of hypernasality for at least six months; 7) Pharyngeal bulb with good retention and comfort; 8) Audio speech recordings in the conditions with and without the pharyngeal bulb with good technical quality; 9) No speech therapy conducted before or after the prosthetic treatment.

Audio recordings from 30 patients, 15 (50%) males and 15 (50%) females were included in this study. These recording were obtained at a mean age of 9 years (ages ranged from 6 to 14 years, SD = 1.87 years), from patients sent to the Speech Appliances Service for temporary use of pharyngeal bulb. Surgical correction of velopharyngeal insufficiency was postponed due to the lack of vacancy for surgery at the time.

### Pharyngeal bulb prosthesis

**Figure 1:**
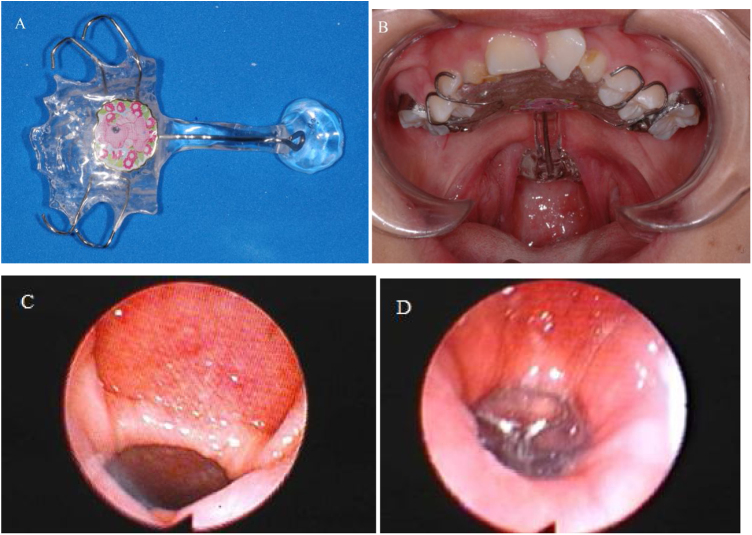
(A) View of the pharyngeal bulb prosthesis; (B) Intraoral view of the pharyngeal bulb prosthesis in place; (C) Nasoendoscopic view of the velopharyngeal gap without pharyngeal bulb prosthesis; (D) Nasoendoscopic view of the velopharyngeal gap with pharyngeal bulb prosthesis.

All participants in this study were fitted with a pharyngeal bulb prosthesis, fabricated by the same dentist in partnership with the same Speech-Language Pathologist (SLP), using a heat-cured acrylic resin.^34^ The final impression of the bulb was established during diagnostic speech therapy, using nasoendoscopic biofeedback (Olympus ENF-P4) of VP function, which provided the dentist and SLP with a superior view of the VPM during speech production. In our service, at the moment of nasoendoscopy, diagnostic therapy during the impression of the bulb in all patients is performed in order to stimulate them to produce oral consonants with no compensatory articulation. In doing so, SLP does not expect VP closure (since the bulb is still under construction), but does expect to see a better movement of the pharyngeal walls and where in the pharynx this movement occurs in order to fit the bulb. For this purpose, the SLP uses strategies for the manipulation of intraoral air pressure during speech, aiming to increase the displacement of the pharyngeal walls (Fig. 1).

### Audio recordings and speech stimuli

Speech recordings were made in a sound-treated room, with the patient seated on an office chair. An AKG C-420 unidirectional condenser microphone was positioned approximately 10 cm from the patient´s mouth. The recordings were made onto a computer with a Creative Audigy II sound card using the Sony Sound Forge program (version 7.0), with sampling rate of 44,100 Hz and a signal resolution of 16 bits. Files were saved in *.wav format. The following set of sentences in Brazilian Portuguese was used for the speech recordings, repeated after the examiner “*Papai caiu da escada* (Dad fell off the ladder), *Fábio pegou o gelo* (Fabio took the ice), *O palhaço chutou a bola* (The Joker kicked the ball), *Tereza fez pastel* (Tereza made pastry), *and A árvore dá frutos e flores* (The tree bears fruits and flowers)”. Patients were audio recorded twice, first without the pharyngeal bulb and then with the device in place.

### Speech recordings editing

The audio recorded pairs of the sentences set (with and without pharyngeal bulb) for all participants were randomly mixed and coded with the letters A and B, which meant that the rater did not know which samples (with or without pharyngeal bulb) came from either letters A or B. In addition to the original 30 pair of recordings containing (30 sentences sets recorded with pharyngeal bulb and 30 recorded without pharyngeal bulb of each participant), 20% (6 pairs of sentences sets) of the recordings were randomly duplicated for intra-rater comparison. The edition was made using the Sound Forge Program 10.0 software and transferred to a CD.

### Auditory-perceptual rating

Three female-certified SLPs who were native speakers of Brazilian Portuguese served as raters. They all had over 5 years of experience with auditory-perceptual speech assessments of speakers with cleft palate. The raters were not aware of the purpose of the study nor were they familiar with any of the speakers who were included as participants. The raters were also blinded to the conditions of the speech samples they were rating, i.e., whether the recordings were done with or without the pharyngeal bulb prosthesis. Hypernasality was rated as present (rating score 1) or absent (rating score 0), based on recordings of the sentences set.

Prior to the actual rating, a 1 h training session was conducted in which the raters rated speech samples representing normal and hypernasal resonances using training samples of patients who were not participants in this study. During this training session, the raters were allowed to discuss ratings and ask questions. They were also instructed orally how to use the rating forms. Printed instructions were provided for them.

All ratings were made independently in a quiet room, with the raters using their own personal computers. They were instructed to listen to both samples (A and B) of each pair of sentences set and rate if hypernasality was present or absent and then write their answer in the rating form as the following:

Pair number:

( ) Sample A with hypernasality and sample B without hypernasality.

( ) Sample A without hypernasality and sample B with hypernasality.

( ) Samples A and B with hypernasality.

( ) Samples A and B without hypernasality.

### Reliability

To establish inter-rater´s agreement, the ratings were compared between the 3 SLPs. inter-rater´s percentage agreements for each pair of raters for the samples without and with bulb were, respectively: R1 vs. R2 = 93% and 97% (κ = 0.63 and κ = 0.92), R1 vs. R3 = 97% and 83% (κ = 0.84 and κ = 0.61), and R2 vs. R3 = 97% and 80% (κ = 0.78 and κ = 0.55).

Interpretation of Kappa (κ) scores can be done according to Landis and Koch (1977),^35^ in which Poor = κ < 0.00; Slight = κ: 0.00‒0.20; Fair = κ: 0.21‒0.40; Moderate = κ: 0.41‒0.60; Substantial = κ: 0.61‒0.80; Almost perfect = κ: 0.81–1.00.

### Data analysis and statistical analysis

Perceptual ratings of hypernasality (presence/absence), with and without pharyngeal bulb prosthesis, were calculated using the Wilcoxon test, considering the equal results of all raters or of at least two raters. Ratings of zero are considered speech recordings with no hypernasality and ratings of 1 speech recordings with presence of hypernasality. The level of significance was set at 5% (*p* < 0.05).

## Results

### Perceptual ratings

**Figure 2:**
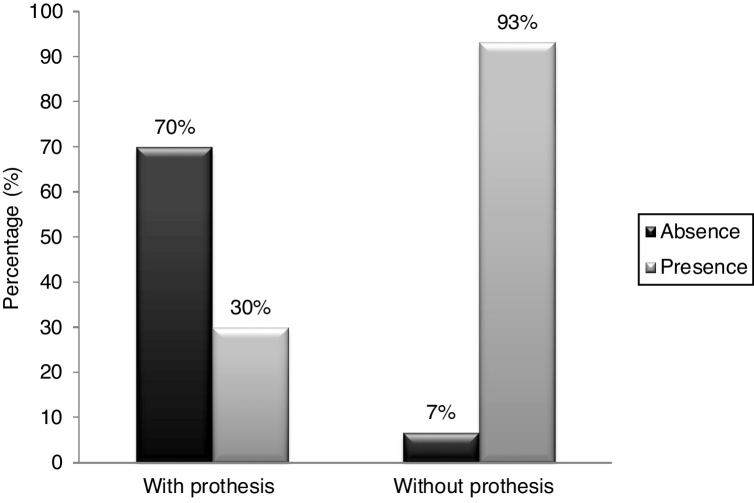
Rater´s judgment of the presence and absence of hypernasality, with and without pharyngeal bulb prosthesis.

Out of the 30 participants, 28 (93%) were rated as presenting with hypernasality and 2 (7%) with no hypernasality when they were not using the pharyngeal bulb. With the pharyngeal bulb in place, 21 (70%) were rated with no hypernasality and 9 (30%) with hypernasality. Paired observations of nasality ratings for speech produced with and without prosthesis showed that a significant reduction in nasalization was associated with obturation. With the prosthesis in place, 19 (63%) participants eliminated hypernasality, 9 (30%) still remained with hypernasality, and 2 (7%) were rated not to present with hypernasality, either with or without pharyngeal bulb prosthesis. The difference between the results was statistically significant (*p* < 0.001), according to the Wilcoxon test, showing a significant improvement in speech resonance with the use of a pharyngeal bulb prosthesis (Fig. 2).

## Discussion

The present study aimed to investigate the outcome of treatment of hypernasality with pharyngeal bulb prosthesis in patients with history of cleft palate presenting with velopharyngeal insufficiency after primary palatal surgery. We hypothesized that the use of the pharyngeal bulb prosthesis is an effective approach to eliminate hypernasality related to velopharyngeal insufficiency in patients with cleft palate.

The results have shown that hypernasality was eliminated for the majority of the patients, which is consistent with results other studies found.^13^, ^15^, ^36^, ^37^ One study found a significant improvement in speech intelligibility (with exception of compensatory articulation) for patients presenting with VPI with the pharyngeal bulb prosthesis in place.^15^ Other studies reported different rating levels of normal resonance when their patients had their VP gaps obturated with a pharyngeal bulb prosthesis: 63%,^13^ 74%,^38^ 90%,^39^ 92%,^13^ and 100%.^40^ However, when analyzing the results obtained in these studies there is the influence of characteristics other than the effect of a pharyngeal bulb prosthesis on speech, such as performing speech therapy to eliminate compensatory articulation and inclusion of patients with different etiologies of VPD, not only congenital cleft palate.

Approximately one third of patients of the present study did not eliminate hypernasality. This failure rate corroborates the findings of the study of Sell, Mars and Worrel.^13^ Understanding the reasons of the speech failure with prosthesis is not easy. Several factors isolated or in complexly interrelated combinations, could explain this failure, such as: persistent compensatory articulation after prosthetic fitting, failure of the pharyngeal bulb to adequately close velopharyngeal gap, presence of severe dysphonia, or other individual structural or functional features that compromise successful prosthetic construction. It has been reported that the presence of dysphonia, especially those related to voice quality and weak vocal intensity parameters, can mask the judgment of nasality.^1^, ^40^ We suggest that future studies control for the presence of these alterations, as they may actually interfere with hypernasality judgment.

In the present study, successful treatment with temporary pharyngeal bulb prosthesis was considered only if hypernasality was eliminated. However, some studies have considered mild hypernasality as an acceptable result of the physical procedure since this deviation is not perceived by the patient and their family and friends.^41^

According to the raters of our study, paired observations of nasality ratings for speech produced for the participants’ nº 11 and nº 16 showed that they did not present hypernasality either with or without prosthesis. Perhaps both of them presented mild hypernasality in the without- prosthesis condition and normal resonance or still mild hypernasality in the with-prosthesis condition, which were not audible for the raters. Recorded speech samples were suggested to solve the limitations of live auditory-perceptual evaluation.^2^, ^42^, ^43^ It is a resource that always can be on hand to be used when and as often as necessary, allowing validating the clinical findings obtained during the live assessment and obtaining intra- and inter-judge reliability measurements, which contributes to increased scientific credibility of the results. However, some disadvantages should be considered, such as: it can capture ambient noise or might not detect enough acoustic information to allow the rater to identify the presence of weak intraoral pressure, mild hypernasality, inaudible air leakage and errors related to speech production.^44^, ^45^ Normal resonance was also found in one study^46^ in which the assessment was conducted by “blind” listeners who judged the absence of hypernasality preoperatively for some patients. Identifying the hypernasality at the extremes of the range (normal vs. severe hypernasality) is an easier task than identifying a light or inconsistent hypernasality, which is not always captured in the recording.^46^

One limitation of the present study may be related to the fact that the auditory perceptual assessment was only adopted for the analysis of VP function with and without pharyngeal bulb prosthesis. In addition to the perceptual judgment, the literature also indicates the instrumental assessment for the diagnosis of VPD and the determination of treatment effectiveness.^1^, ^2^, ^47^ Also, hypernasality (speech resonance) was the only parameter analyzed. For future studies we suggest the inclusion of an instrumental assessment of VP function to compare the results with and without temporary pharyngeal bulb prosthesis, as well the assessment of speech articulation and intelligibility, voice, and the occurrence of nasal air emission, in order to investigate the variables that can influence the non-elimination of hypernasality using the prosthesis.

The use of a temporary pharyngeal bulb prosthesis may have identified possible candidates for secondary surgery with a good prognosis for speech, specifically the elimination of hypernasality and adequacy of VP function and, likewise, have identified patients who would not benefit from surgery at the present time. Such identification was performed in some studies in which the pharyngeal bulb prosthesis was temporarily successful before the surgical procedure.^13^, ^47^

## Conclusion

The use of the pharyngeal bulb prosthesis is an effective approach to eliminate hypernasality related to velopharyngeal insufficiency.

## Conflicts of interest

The authors declare no conflicts of interest.
